# The psychology of Mona Lisa’s smile

**DOI:** 10.1038/s41598-024-59782-1

**Published:** 2024-05-28

**Authors:** Alessandro Soranzo

**Affiliations:** Laboratory of Modelling, Simulation, and Visualization (LMSV), Physics Department, University of Calabria (ITALY), Cubo 17/B, Ponte Bucci, 87036 Rende, CS Italy

**Keywords:** Mona Lisa, Perceptual organisation, Facial expression, Ambiguity-Nuance, Neuroscience, Psychology

## Abstract

Mona Lisa’s ambiguous expression, oscillating between melancholy and contentment, has captivated viewers for centuries, prompting diverse explanations. This article proposes a novel interpretation grounded in the psychological theory of perceptual organisation. Central to the investigation is the “Ambiguity-Nuance”, a subtly shaded, blended region framing the upper part of the lips, hypothesised to influence perceived expression due to perceptual organization. Through carefully crafted artwork and systematic manipulations of Mona Lisa reproductions, experiments reveal how alterations in the perceptual relationships of the Ambiguity-Nuance yield significant shifts in perceived expression, explaining why Mona Lisa’s appearance changes and under which conditions she looks content versus melancholic based on perceptual organization. These findings underscore the pivotal role of psychological principles in shaping ambiguous expressions in the Mona Lisa, and extend to other Leonardo’s portraits, namely La Bella Principessa and Scapigliata. This study sheds light on the intersection of psychology and art, offering new perspectives on timeless masterpieces.

Mona Lisa’s (Fig. 1) ever-changing expression is one of the most enchanting aspects of Leonardo da Vinci’s iconic masterpiece^1^ (p. 12:259). The subtle ambiguity of her smile has fascinated generations of viewers and scholars, who have proposed various interpretations of this phenomenon. Some have argued that ambiguity is an intentional trompe l’oeil effect, due to a magistral manipulation of light and shadow to create an illusion of movement^2^. Others see it as a masterful depiction of human emotional duality, simultaneously embodying happiness and melancholy, inviting endless subjective interpretations^3^.

**Figure 1:**
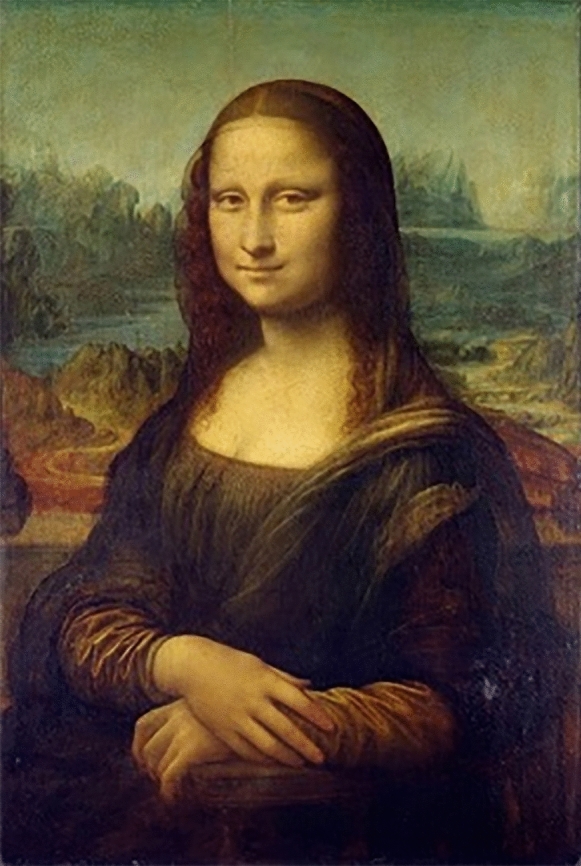
Mona Lisa (1503–1506), Louvre. *Source*: https://www.wikiart.org.

However, none of these interpretations consider the psychological theory of perceptual organisation^4^. This article proposes a novel interpretation based on this psychological theory. It is argued that Mona Lisa’s expression is determined by the way visual features are perceptually organised. Specifically, the focus is on how the artistically crafted darkened blurred region framing the upper part of the lips, defined here as the “Ambiguity-Nuance”, perceptually organizes with the mouth.

This interpretation builds upon Livingstone’s account^5^ of the dynamic interplay between *melancholy* and *contentment*.

[The Mona Lisa is renowned for her ambiguous smile^1,5^. A noun like “smile” can lead to the hasty assumption that perceived expression is related to the happy/sad emotional continuum, as certain authors have claimed^6,7^. However, Mona Lisa’s expressions may not pertain to emotions but to moods. Giorgio Vasari’s account of Leonardo’s ploy suggests that musicians and clowns were employed to make the Mona Lisa *merry* and alleviate her *melancholy*^8^. These adjectives refer to a state of mind, not to an externally visible expression: moods, not emotions. This aligns with Leonardo’s concept of *moti mentali*, introduced in his Trattato della Pittura^9^, which aims to represent inner thoughts and dynamic mental states rather than outward emotions. Finally, qualitative investigations suggest that the ambiguity in Mona Lisa’s expression is indeed better captured by the contentment/melancholic dimension^10^].

Livingstone suggests that scrupulous use of *sfumato* contributes to the apparent change of expression. *Sfumato* is a painting technique in which a translucent layer of paint is laid over an opaque one, generating an overlay of multiple coatings. In this way, the transitions from bright to dark, or from one colour to another, are subtle, softening, or obscure sharp edges^11^.

Livingstone interprets the effects of *sfumato* on expression change focussing on the properties of the retinal receptors. Our retina contains two types of photoreceptors: cones, gathered in the central region, which detect minute details, and rods, located in the peripheral region of the retina, which can only capture coarse aspects of visual stimuli. According to this interpretation, ambiguous expression arises from eye movements. When we direct our gaze to Mona Lisa’s mouth, the cones, capable of discerning minute details, lead us to perceive a melancholic expression. Conversely, as our gaze shifts to other areas of the painting, the rods, which capture only broad aspects of the image, register Mona Lisa’s mouth without capturing its minute details—since they are blurred by *sfumato*—leading to a perception of contentment. Therefore, dynamism and ambiguity derive from a constant switch between melancholy and contentment, detected by cones and rods, respectively.

Although this interpretation brings clarity to the systematic nature of the phenomenon (it is the visible details of the mouth that determine the perceived expression, not the viewer’s imagination or state of mind, Livingstone explained^12^), it does not entirely address why melancholy is perceived when minute details are clearly visible, whereas contentment emerges when the details are unclear. There would be a dynamic effect even if contentment were detected by cones and melancholy by rods. However, this is not the case.

This raises a fundamental question: *Why do we perceive contentment when the details of Mona Lisa’s mouth are unclear and melancholy when they are clear? Why not the other way around?*

The legitimacy of this question finds support in the presence of a similar expressive dynamism in other artworks attributed to Leonardo da Vinci, such as La Bella Principessa (Fig. 2, left^10^) and the Young Woman with Tousled Hair or Scapigliata (Fig. 2, right^13^). Like the Mona Lisa, their expression fluctuates between contentment and melancholy.

**Figure 2:**
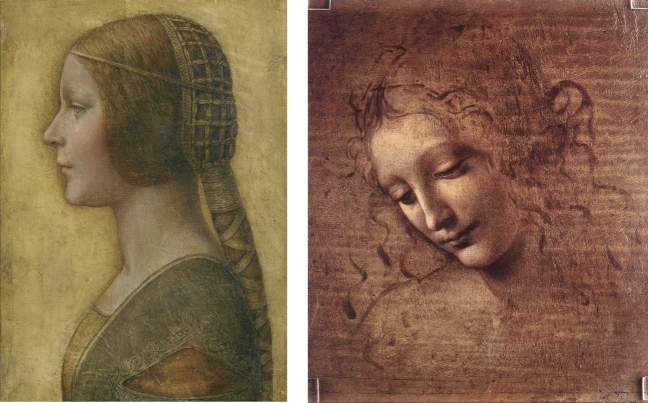
(Left) La Bella Principessa (1495–1496, private collection); (Right) Young Woman with Tousled Hair or Scapigliata (1508, Galleria Nazionale, Parma). *Source*: https://www.wikiart.org.

[Both masterpieces are credited to Leonardo, but not unanimously. The paternity of these artworks is irrelevant to this project. It is more relevant, instead, that their expression changes in the same way as in the Mona Lisa].

What deserves emphasis is not only that these portraits exhibit an ambiguous expression but also the consistent pattern of contentment being perceived when mouth details are unclear and melancholy when mouth details can be discerned.

This is no coincidence. The mouth borders of these portraits are painted with greater attention than any other area of the paintings^14,15^. In particular, the outline of the mouth is painted with such dedication that it seamlessly blends with the surrounding facial areas. In this way, the separation between the mouth and contiguous areas is only apparent on close examination. This observation invites a broader perspective that extends beyond the mouth. Looking beyond the mouth, a notable feature emerges: a subtly shaded blurred region framing the upper part of the lips, created through Leonardo’s masterful use of *sfumato*. This is the Ambiguity-Nuance.

Both Mona Lisa and Scapigliata exhibit two Ambiguity-Nuances, one at each corner of the mouth. La Bella Principessa, shown in profile, features only one Ambiguity-Nuance. This element, both shadowy and mouth-like, becomes the focal point of this study. When examining the Ambiguity-Nuance, Gestalt theory becomes especially relevant.

Gestalt theory, advanced by psychologist Max Wertheimer in the early 1900s, builds on perceptual organisation principles to explain how our brain combines distinct elements into cohesive objects. For instance, when observing a window in a house across the street, we see it as one object, even if partially obscured by a tree branch or unevenly lit owing to shadows from the roof. Despite being fragmented and uneven, our brain organises these visual inputs into a unified window. This process relies on perceptual organisation principles.

A key principle for this study is “good continuation”. It suggests that elements with smooth and unbroken contours are seen as single objects^4^. In the house window example, we see it as a unified window, even if a tree branch partially obscures it, because our brain assumes that it continues behind the branch and reappears on the other side. This tendency to see objects continuing in their expected direction defines “good continuation”. Significant to this study is also the “common region” principle^16^. It posits that elements within the same enclosed region are perceived as a unified object. Returning to our example of the window in a house across the street, we perceive it as a cohesive window despite uneven lighting because the different light intensities fall within the same “common region”—the confines of the window frame.

Applying these principles to the Ambiguity-Nuance and the mouth leads to the following hypothesis:

When minute details are not readily visible, the distinction between the mouth and the nuance becomes less noticeable. With “good continuation”, the Ambiguity-Nuance appears to merge with the mouth, resulting in a gentle smile. (This aligns with anthropological research indicating that smiles typically involve upturned lip corners^17,18^).

In contrast, when the details are discernible, a boundary is visible between the mouth and the nuance. Under these conditions, the mouth and the Ambiguity-Nuance are perceived as distinct elements. The mouth is enclosed within a “common region”, defined by its borders, and the Ambiguity-Nuance appears as a shadow on the cheek, not affecting the perception of the mouth’s slant.

To empirically assess this hypothesis, two essential steps are undertaken. First, expressive ambiguity is defined quantitatively. Drawing on Livingstone’s interpretation that expression changes with visible details^6^, ambiguity can be conceptualised by assessing perceived expression in relation to visible details; with an expression deemed ambiguous when it changes with variations in visible detail. This method of assessing ambiguity ensures a more robust and reliable estimate than directly asking participants to gauge an expression’s ambiguity. The second critical step involves manipulating visible details to study their impact on perceived expression. In accordance with Livingstone’s observation, one way to achieve this could be by varying the eye position, but this method is impractical. A more feasible way is to manipulate the viewing distance. At a distance, minute details become undetectable because they are compressed. This choice was validated by a pilot experiment, revealing a notable change in perceived expression when moving from 0.6 to 6 m in viewing distance. [Ten out of ten observers perceived a discernible variation in the Mona Lisa’s contentment when viewed from the two distances. Furthermore, her expression was reported as being perceptible even from afar. None of these initial respondents participated in the subsequent experiments].

Two experiments were conducted to assess the hypothesis that the changes in expression observed in Mona Lisa, Bella Principessa, and Scapigliata stem from the perceptual organisation of the Ambiguity-Nuance. This was achieved by creatively manipulating portraits reproductions, wherein digital images of each portrait were altered by artists employed as part of the project, and the modified images were then printed for experimentation.

In Experiment 1, the portraits were manipulated by drawing a thick line around the lips (Fig. 3).

**Figure 3:**
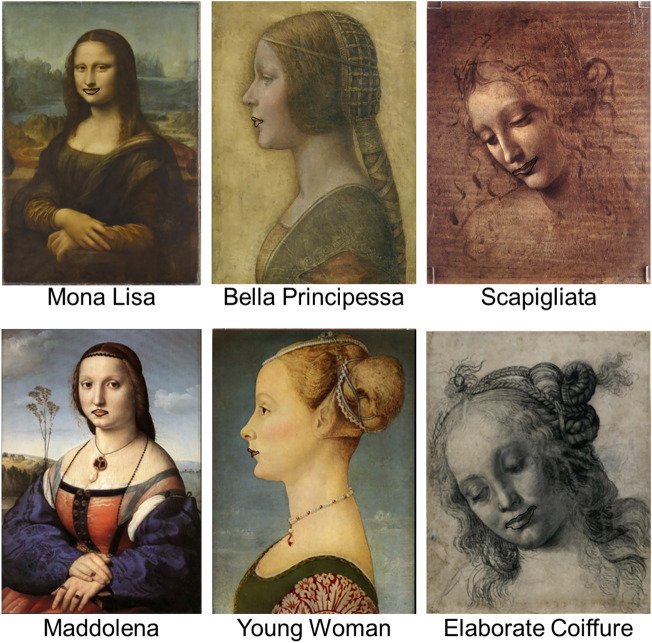
Representation of stimuli used in Experiment 1 under the condition with the line. The first row shows the experimental portraits, and the second row shows the matching controls. *Source*: Mona Lisa, Bella Principessa, Scapigliata and Maddolena, adapted from https://www.wikiart.org. Young Woman and Elaborate Coiffure, adapted from https:/wikipedia.org.

[Figures are added for the sake of clarity. The quality and size of the images might not render the perceptual effect that is observable in the printed, high-quality, actual size images that were used in the project].

The rationale for this experimental manipulation is as follows: If contentment increases when fewer details are visible because the Ambiguity-Nuance merges with the mouth (as hypothesized), then contentment should not increase if this merging process is prevented. The thick line is expected to impede this process for two reasons: (1) it interrupts the ‘good continuation’ by creating a clear discontinuity between the mouth and the Ambiguity-Nuance, and (2) it encloses the mouth within the same ‘common region,’ reinforcing the perception that the mouth is separated from the Ambiguity-Nuance.

To assess the selective impact of this manipulation on portraits featuring the Ambiguity-Nuance, three control portraits were included in the study. These control portraits were carefully chosen to match the artistic period, size, presentation, and subject matter of their respective counterparts. Specifically:

- The portrait of Maddolena Doni (c.1506) by Raffaello Sanzio da Urbino was chosen as a suitable control for the Mona Lisa; the. This artwork was created just a few years later than Mona Lisa by a renowned Renaissance master. Its half-length format, contemplative sitter pose, and muted colour palette provide stylistic parallels. As the wife of a wealthy Florentine, Maddolena’s portrayal also reflects the societal standing of Mona Lisa.
- The portrait of a Young Woman (c. 1470) by Piero del Pollaiuolo was chosen as a suitable control for La Bella Principessa; the. The two portraits are in profile and share a similar soft shading of the sitters’ features. The Young Woman’s aristocratic dress and elaborate coiffure parallel the elite status implied by the sitter of La Bella Principessa.
- The Woman with Elaborate Coiffure (c.1480) by Il Verrocchio was chosen as a suitable control for the Scapigliata. Both depict fashionably dressed women in pensive poses with loose, flowing hairstyles. Verrocchio’s skilled portrayal of flowing drapery and naturalistic hair also matches Scapigliata’s depiction.

Drawing precise lines in the experimental portraits posed a significant challenge. Within these artworks, distinguishing the boundary between the mouth and the Ambiguity-Nuance proved difficult, even upon close examination. The artists relied on their own artistic flair to address this challenge, and it is conceivable that different artists may have approached the task with slight variations, potentially resulting in different expressions. However, such potential variations were inconsequential to the objectives of this study. The primary purpose of the drawn lines was to maintain a consistent level of contentment, ensuring that perceived contentment remained unchanged. The specific level of contentment perceived at each distance held no relevance to the focus of this study.

In Experiment 2, the portraits underwent manipulation by relocating the Ambiguity-Nuance from above to below the corners of the mouth (Fig. 4). The rationale for this experimental adjustment is as follows: If contentment increases when fewer details are visible because the Ambiguity-Nuance merges with the mouth (as hypothesized), then contentment should decrease if the nuance causes the mouth to appear downward. This is because a downturned mouth tends to convey melancholy^18^.

**Figure 4:**
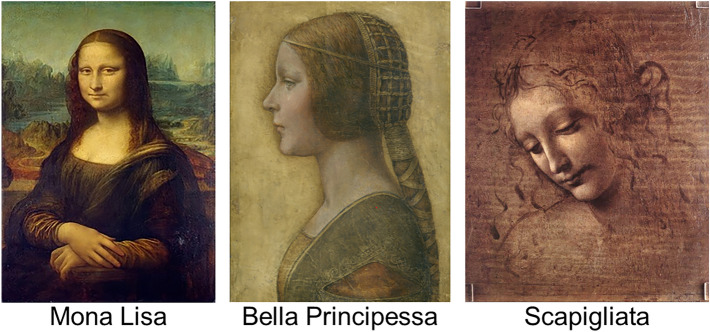
Representation of stimuli used in Experiment 2 under the condition Ambiguity-Nuance below the corners of the mouth. *Source*: adapted from https://www.wikiart.org.

Furthermore, it can be hypothesized that if contentment changes due to the perceptual organization of the Ambiguity-Nuance, rather than simply its presence, contentment should remain consistent from close-up, irrespective of its position. When viewed closely, the boundary between the mouth and the nuance remains distinguishable, and the nuance should consistently appear as a shadow, not connected to the mouth. Therefore, this should have no impact on the perceived expression.

In each experiment, thirty-two participants were randomly divided into two viewing distance groups. Participants were instructed to rate the level of contentment—defined as “the feeling of quiet and internal satisfaction”—on a scale of 1 (no content, or melancholy) to 7 (very content). (see Method for details about stimuli, sample size, participants and procedure).

The following variables were systematically manipulated:

1. Viewing Distance: Close, 0.6 m and Far, 6 m;
2. Portraits: Mona Lisa, La Bella Principessa and Scapigliata (plus their respective controls in Experiment 1);
3. Line: Present and Absent (in Experiment 1)—Ambiguity-Nuance Position: Below versus Above (in Experiment 2).

## Results

For both experiments, a Bayesian mixed-effects model was run with a cumulative distribution linked to the probit function^19,20^. Of particular relevance to this study is the planned post hoc comparison, which effectively illustrates the impact of visible details on each portrait. (A comprehensive statistical analysis is available in the Supplementary file.)

**Figure 5:**
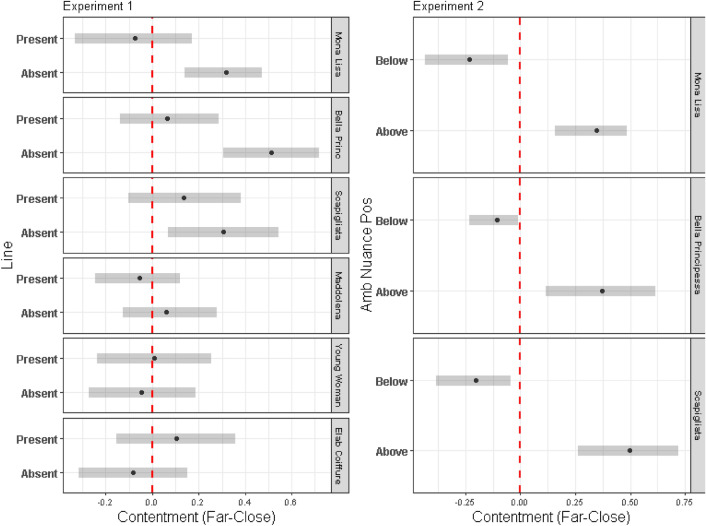
Medians (black dots) and Highest Density Intervals (HDI, horizontal grey bars) of the estimated differences between the two distances obtained for experiment 1 (left) and experiment 2 (right). The HDI bars that include zero (crossed by the vertical red dashed line) signify that contentment is practically the same in the Close and Far conditions.

Figure 5 displays the relative positions of the medians (indicated by black dots) and Highest Density Intervals^21^ (HDIs, indicated by horizontal grey bars) of the estimated differences between the two viewing distances.

[Highest Density Intervals are similar to confidence intervals but provide a range of probabilities for a parameter, rather than a fixed confidence level. A highest density interval differs from a confidence interval in that all points within the interval have a greater likelihood than any points outside the interval].

The HDI bars that intersect with zero (crossed by the vertical red dashed line) indicate that the perceived contentment remains practically unchanged between the close and far viewing distances.

In Experiment 1, as depicted in Fig. 5 (left), only the HDI bars associated with Mona Lisa, Bella Principessa, and Scapigliata without the line do not encompass zero, indicating an ambiguous expression with a notable change between close and far distances. Specifically, these bars are positioned on the positive side of the graph, signalling increased contentment when fewer details were visible from a distance. In contrast, the HDI bars of the experimental portraits with a line, as well as in the control portraits (both with and without a line), encompass zero. This suggests that in these cases, contentment remains consistent regardless of mouth visibility; hence, no expressive ambiguity was recorded in these portraits. These findings support the hypothesis that changes in expression are influenced by the perceptual organisation of the Ambiguity-Nuance. The line around the mouth prevents the nuance from merging with the mouth, thus halting the change in expression.

It is noteworthy that the control portraits did not demonstrate a difference in contentment with changes in viewing distance irrespective of the line around the mouth. This underscores the uniqueness of experimental portraits.

In Experiment 2, as depicted in Fig. 5 (right), none of the HDI bars encompass zero, indicating a substantial difference in contentment under all conditions. Notably, an interesting pattern emerges: While contentment increased with distance in the unmodified portraits (the HDI bars are on the positive side of the graph), it decreased with distance in the modified portraits (the HDI bars are on the negative side of the graph). This reversal effect stands in contrast to the original transformation.

**Figure 6:**
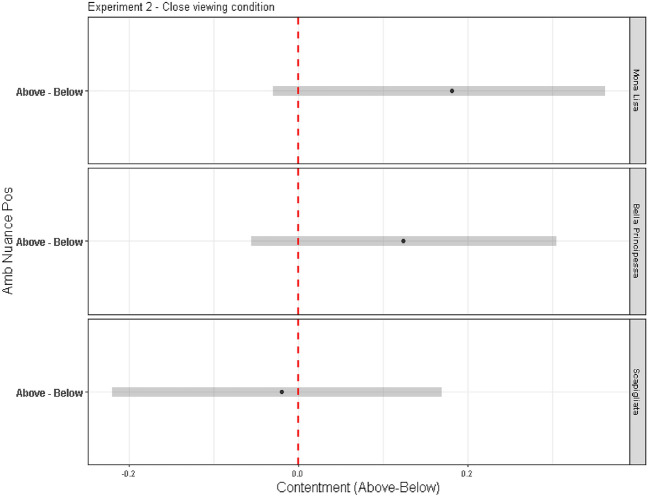
Medians (black dots) and Highest Density Intervals (HDI, horizontal grey bars, see note 5) of the difference in contentment between the above and below Ambiguity-Nuance conditions from close.

Experiment 2 yielded an additional noteworthy result. Figure 6 shows the estimated differences between contentment recorded in portraits with the Ambiguity-Nuance above the mouth and below the mouth when viewed up close. Interestingly, for all portraits, the HDI bars encompass zero, signifying a similar level of contentment whether the Ambiguity-Nuance was above or below the mouth. This outcome can be explained by recognising that, when viewed closely, the distinction between the mouth and the nuance becomes discernible. This clear demarcation causes the mouth and nuance to appear as separate elements. Consequently, this finding underscores the hypothesis that it is the perceptual organisation applied to the Ambiguity-Nuance that induces a change in expression, rather than the mere presence of the nuance.

## Discussion

Mona Lisa’s ambiguous expression has fascinated audiences for centuries. At times she conveys contentment, while at others, a veil of melancholy darkens her demeanour^1^. This article offers an original interpretation of this ambiguity rooted in psychological theory.

This interpretation builds upon Livingstone’s work^5^ on how *sfumato* contributes to the ambiguous expression, suggesting that the interplay of expression relies on retinal receptors. Melancholy is evoked when the mouth is observed with central receptors, while contentment is evoked by peripheral receptors. Livingstone’s theory does not fully explain why melancholy appears when mouth details are seen and contentment when they are not. An ambiguous expression would emerge even with melancholy characterized by unclear details and contentment by clear details. However, this is not the case. A smile consistently appears more evident when details are unclear.

(Somehow paradoxically, the Mona Lisa’s renowned smile becomes more pronounced precisely when it escapes clear perception).

The phenomenon of ambiguous expression extends beyond the Mona Lisa to other masterpieces. Portraits like La Bella Principessa^10^ and the Scapigliata^13^ consistently evoke contentment when details are unclear and melancholy when they are discernible. To fully comprehend this phenomenon, analysis must extend beyond the mouth. A psychological approach based on Gestalt theory can provide invaluable insight into the holistic nature of perception. Within these portraits, the boundary between the mouth and the subtly shaded, blended region framing the upper part of the lips becomes apparent only upon close examination. This region is designated Ambiguity-Nuance because it elicits a dual perception, being shadowy and mouth-like at the same time.

The central hypothesis of this research posits that ambiguous expression hinges on the perceptual organisation of the Ambiguity-Nuance. It is suggested that this feature shapes the expression based on its perceptual organisation and the visibility of mouth details. When fewer details are visible, the boundary between the Ambiguity-Nuance and the mouth becomes imperceptible. In this scenario, the perceptual principle of “good continuation”^4^ causes the nuance to merge with the mouth. As the Ambiguity-Nuance stretches from the upper part of the lips, this process imparts an upward direction to the mouth, ultimately creating the impression of a gentle smile.

Conversely, when the boundary between the mouth and the Ambiguity-Nuance is clear because of enhanced detail visibility, the mouth and nuance appear as distinct entities. In this case, the mouth is enclosed within the same “common region”^16^, and the nuance looks like a shadow on the cheek. This does not affect the perceived slant of the mouth, which is not conducive to smiling or contentment.

To empirically assess this hypothesis, two experiments were conducted using newly created artworks, where the Ambiguity-Nuance and mouth visibility were manipulated. These experiments provided crucial insights into the nuanced interplay between perceptual organisation and the portrayal of ambiguous expressions in these works of art. Experiment 1 demonstrated that introducing a thick line to prevent the merger of the Ambiguity-Nuance with the mouth halted dynamic expression changes, underscoring the role of an unclear border between the Ambiguity-Nuance and the mouth in driving expression changes. Experiment 2 showed that when the Ambiguity-Nuance was shifted below the mouth, melancholy was enhanced with unclear details—an effect opposite to the original, where melancholy decreased with unclear details. Importantly, repositioning the nuance had no impact on perceived expression when the details were clear (from up close), indicating that the Ambiguity-Nuance influenced expression only with unclear details.

Taken together, these findings demonstrate the inherent value of a psychological explanation grounded in perceptual organization principles for understanding the ambiguous expressions of the Mona Lisa, La Bella Principessa, and Scapigliata. Specifically, this explanation reveals why their expressions change and under which conditions they appear melancholic rather than content based on the perceptual relationships between facial features, like Ambiguity-Nuance and the mouth.

This approach not only sheds light on the importance of perceptual organisation, but also opens avenues for further exploration. For instance, there is a suggestion that Mona Lisa’s eyes may also influence perceived expression^22^. Exploring the Ambiguity-Nuance in relation to the eyes, for example, by altering the outer corners, presents a fascinating extension of this study.

Leonardo da Vinci’s deep interest in human cognition and emotion is evident in his artistic pursuits. He aimed not only to depict actions but also to convey the emotions and thoughts driving them. As highlighted in his “Trattato della Pittura”, Leonardo urged portraitists to capture transient and dynamic mental states, known as “moti mentali”^9^, expanding beyond the mere portrayal of external features. Therefore, it is plausible that his deliberate intent extended beyond representing external appearances to represent the subjects’ inner emotional turmoil. The dynamic change of expression observed in the portraits examined here effectively conveys a sense of inner turmoil in the subjects. Leonardo masterfully guided this interplay of expressions, where subjects appear content at first glance, yet a closer look reveals a subtle hint of melancholy. This artistic achievement was realised by skilfully blending the mouth borders and strategically positioning the Ambiguity-Nuance just above its corners. Whether Leonardo consciously applied the psychological principles of perceptual organisation, formally recognised four centuries later, remains to be seen.

## Method

### Stimuli

High-definition digital images used in the experiments were purchased from Alamy (https://www.alamy.com/) and modified by artists employed as part of the project using Adobe Photoshop v21. For Experiment 1, the artists drew a thick line of 14 pixels around the mouth of each portrait. This line width was chosen to be visible from all experimental distances, while leaving the lips visible. For Experiment 2, the Ambiguity-Nuance was digitally relocated from above to below the corner of the mouth in each portrait. These images were then printed using a Canon 10,010 digital colour printer. The prints were of high quality, frameless, and precise size. They were backed with foam and mounted on a wall at a height of 1.80 m from the floor in a room with diffuse lighting, allowing for an unimpeded approach. The white wall behind the portraits had luminance values of approximately 67 and 72 cd/m^2^ under close and far-viewing conditions, respectively.

**Figure 7:**
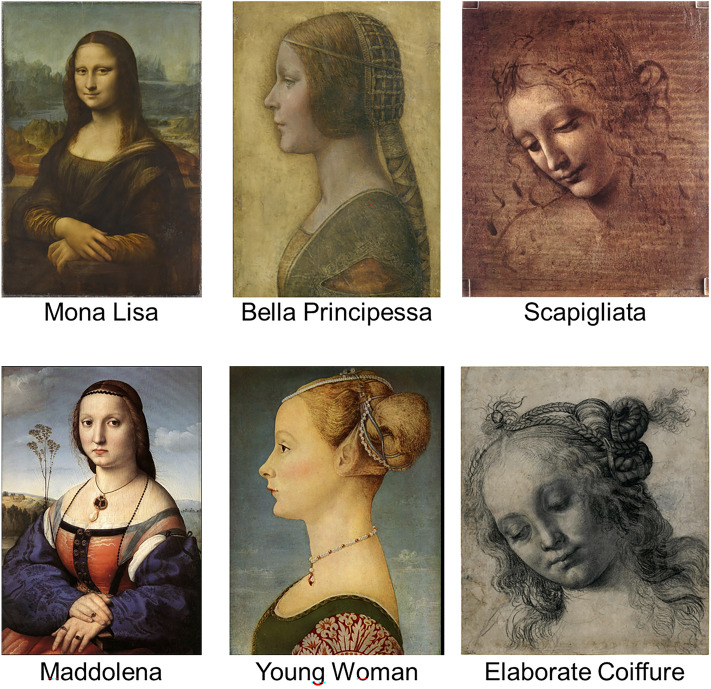
Representation of stimuli and labels used in the project. *Source*: Mona Lisa, Bella Principessa, Scapigliata and Maddolena: https://www.wikiart.org. Young Woman and Elaborate Coiffure: https:/wikipedia.org.

The three portraits displayed in the first row of Fig. 7 were used in both the experiments. Experiment 1 included three further control portraits that are displayed below their corresponding counterparts (see the main section on the selection criteria of these controls).

In both experiments, the first independent variable was the Viewing Distance, which had two levels: Close (0.6 m) and Far (6 m). Table 1 provides the dimensions of the portrait prints (in cm; height × width), as well as the corresponding sizes of the retinal images (in degrees of visual angle) at the two viewing distances. The viewing Distance was manipulated between subjects to control for the “experimental subordination” phenomenon^23,24^. This was done to prevent participants from adjusting their responses based on what they thought they were expected to answer if they had seen the same portrait at different distances. In both experiments, the dependent variable was perceived content, which ranged from 1 (not content) to 7 (very content).

**Table 1:** Sizes and visual angles of the stimulus used in the project.

Portrait	Size	Vis. angle—close	Vis. angle—far
Mona Lisa	77 × 53	65.4° × 47.7°	7.3° × 5.1°
Bella Principessa	33 × 23.9	30.8° × 22.5°	3.1° × 2.3°
Scapigliata	24.7 × 21	2.6° × 19.9°	2.3° × 2°
Maddolena	63 × 45	55.4° × 41.1°	6° × 4.3°
Young Woman	45.5 × 32.7	41.5° × 30.5°	4.3° × 3.1°
Elaborate coiffure	32.5 × 27.2	30.3° × 25.5°	3.1° × 2.6°

### Sample size

Overall, the study involved sixty-four participants, with thirty-two participants in each of the two experiments (sixteen per viewing condition). This figure was supported by an a priori power analysis based on the results of a previous study using a similar experimental design^25^. The pwrss package^26^ run in R version 4.2.3^27^ was used for this purpose. This analysis indicated that a sample size of sixteen participants per viewing condition provided a power of 0.8 for detecting the effect.

### Participants

In Experiment 1, the participants ranged in age from 18 to 48 years and included 21 females and 11 males. In Experiment 2, the age range was 18–54 years, with a participant pool of 27 females and 5 males. All participants had normal or corrected-to-normal visual acuity and were unaware of the study’s purpose. Participants took part voluntarily and received no compensation. Written informed consent was obtained from all the participants in accordance with the ethical protocol of the university.

### Procedure

Portraits were displayed on a wall at the end of a corridor within a designated area on the university campus. The participants were randomly assigned to either the close- or far-viewing conditions. Upon reaching one of the two marked locations on the floor, the participants were asked to rate the level of contentment expressed by the figures in the portraits, which were also presented in random order. Contentment was defined as “a state of quiet and internal satisfaction”. Participants were informed that a rating of 1 corresponded to a low level of contentment or melancholy, whereas a rating of 7 represented the highest level of contentment.

The approximate durations of the experiments were 20 min and 12 min for experiments 1 and 2, respectively.

### Ethical approval and informed consent

This project was approved by the Psychology Research Ethics Panel at Sheffield Hallam University (nr. ER6557404).

### Supplementary Information

Supplementary Information.

## Data Availability

Digital versions of high-quality pictures used as stimuli, data sets and the code for analysis are provided as part of the replication package together with a quarto version of this article here: https://osf.io/zgd3r/.
