# Supplementary material for: The psychology of Mona Lisa’s smile
Source: Sci Rep. 2024 May 28;14:12250. doi: 10.1038/s41598-024-59782-1 (PMC11133332; doi:10.1038/s41598-024-59782-1)
Supplement: Supplementary file 1 — Supplementary Information. [file 41598_2024_59782_MOESM1_ESM.docx]

Supplementary Information:

The psychology of Mona Lisa’s expression

##

## **Corresponding author:**

## Alessandro Soranzo - email: [**alessandro.soranzo@unical.it**](mailto:alessandro.soranzo@unical.it)

## **Affiliation**

## Laboratory of Modelling, Simulation, and Visualization (LMSV)

## Physics Department

## University of Calabria (ITALY)

## Cubo 17/B, Ponte Bucci

## 87036 Rende, Cosenza

## phone: +39 0984 494370

## fax: +39 0984 491011

## **Comprehensive Statistical Analysis**

The data were analysed using Bayesian mixed-effects models. Each participant was assigned a unique intercept for the random structure of the model. These models were created in the Stan computational framework^1^ and accessed using the high-level interface brms package 2.10.0^2,3^ in R version 4.2.3^4^. Data were analysed with ordered-probit models as recommended by Liddell & Kruschke^5^, and the “cumulative” distribution associated with the “probit” link function was implemented in the model specification. To enhance convergence and prevent overfitting, weakly informative priors were set for intercept and population-level effects. The model estimation used four chains with 3,000 iterations and 1,000 warm-ups. Model diagnostics were checked via convergence statistics (Rhat close to or equal to 1.0) and visual inspection of the trace plots^6^.

All credible intervals were the Highest Density Intervals (HDIs)^7–9^. [Unlike confidence intervals that give a single confidence level, Highest Density Intervals provide a range of probabilities for an estimated parameter value. The key distinction is that for HDIs, every value inside the interval has a higher probability of being the true parameter value compared to any value outside the interval bounds.] A decision rule based on HDIs and predefined regions of practical equivalence (ROPE) around zero was applied^10^. Model comparison was conducted by means of the leave-one-out (LOO)^11^ information criterion.

[The score provided by the leave-one-out (LOO) information criterion can be interpreted as other information criteria, such as Akaike’s Information Criterion (AIC)^12^. In this context, smaller values indicate better fit. To determine whether one model was superior to the other, the difference in the LOO information criterion between the two models needed to exceed twice the corresponding standard error^13^.]

Post-hoc comparisons were extracted using the emmeans package^14^. To ensure thoroughness, the results are presented according to Kruschke’s guidelines^15^.

## **Experiment 1**

### Statistical Analysis

[Table 1](#tbl-descrexp1) presents the medians and median absolute deviations (MADs, in brackets) of the levels of contentment recorded in Experiment 1.

[The median absolute deviation (MAD) is a robust measure of variability, similar to the standard deviation but less sensitive to outliers. Unlike the standard deviation which measures the spread from the mean, the median absolute deviation measures the spread from the median, thus used with ordinal data.]

Table 1: Medians and MADs (in brackets) of the levels of contentment recorded in Experiment 1.

| Portrait | Line | Close | Far |
| --- | --- | --- | --- |
| Mona Lisa | Absent | 4(0.74) | 5.5(1.48) |
| Mona Lisa | Present | 5(1.48) | 4(1.48) |
| Bella Principessa | Absent | 3(1.48) | 5(1.48) |
| Bella Principessa | Present | 3(1.48) | 3(1.48) |
| Scapigliata | Absent | 3(1.48) | 4.5(0.74) |
| Scapigliata | Present | 5(1.48) | 5(1.48) |
| Maddolena | Absent | 2.5(2.22) | 3(1.48) |
| Maddolena | Present | 3(1.48) | 3(2.22) |
| Young Woman | Absent | 3.5(2.22) | 4(1.48) |
| Young Woman | Present | 3.5(2.22) | 4(0.74) |
| Elaborate Coiffure | Absent | 4(1.48) | 3.5(2.22) |
| Elaborate Coiffure | Present | 4(1.48) | 4(1.48) |

| 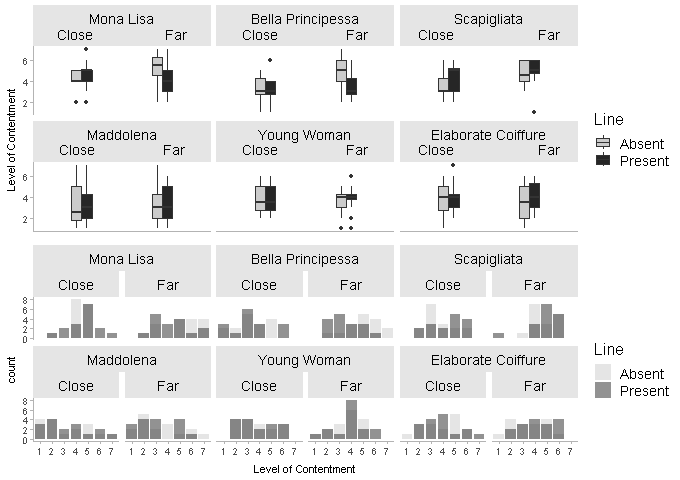  Figure 1: Top two rows: Box plot of contentment for the three experimental portraits and the three control portraits used in Experiment 1. Bottom two rows: Frequency of the seven ratings for the same portraits. |
| --- |

The first two rows of [Figure 1](#fig-resExp1) display the box plot of the contentment level, and the last two rows show the frequency of the seven ratings organised by Portrait and Viewing Distance of Experiment 1.

As seen in the first two rows of [Figure 1](#fig-resExp1), the light bars (indicating unmodified portraits) are higher in the Far condition than in the Close condition for experimental portraits, whereas the dark bars (indicating portraits with lines) remain approximately the same across all conditions.

In the last two rows of [Figure 1](#fig-resExp1), the light bars (indicating unmodified portraits) are more frequent in the lower range of contentment in the Close condition than in the Far condition. However, this holds only for experimental portraits. The dark bars (indicating portraits with lines) are evenly spread across the contentment range for the other portraits.

This shows that, while perceived contentment increased with distance in the unmodified portraits, it did not change in the other portraits.

### Modelling

A set of Bayesian linear mixed-effects models was used. Supplementary [Table 2](#tbl-selection_Exp1) shows the results of leave-one-out (LOO)^11^, which is the information criterion used to select the model with the best predictive abilities.

Table 2: Model selection.

| **Model** | **LOO diff** | **st.error** |
| --- | --- | --- |
| Portrait | 0.0 | 0.0 |
| Distance*Portrait | -0.5 | 3.2 |
| Line*Portrait | -1.8 | 3.2 |
| Line*Distance*Portrait | -2.7 | 6.5 |
| Distance | -18.4 | 7.4 |
| Line*Distance | -18.6 | 7.7 |
| Empty model | -18.6 | 7.4 |
| Line | -19.8 | 7.5 |

As shown in [Table 2](#tbl-selection_Exp1), models that include Portrait have similar predictive abilities. This shows that the effects of Line and Viewing Distance were similar for all the portraits. Indeed, the effects of Line and Viewing Distance were expected to differ only for the experimental portraits without lines. The leave-one-out (LOO) information criterion of the model that contains only Portrait is 1301.7 (standard error = 23.5).

Nevertheless, in order to examine the effects of Line and Viewing Distance on perceived contentment separately for each portrait, which is the aim of this study, a full model that also incorporates Line and Viewing Distance is required.

The LOO of the full model is 1307.1 (standard error = 24.9), which is similar to the LOO of the model that contains Portrait only. The subsequent post hoc analysis was based on the full model.

Category-specific and unequal variance models were run using the acat() function instead of the cumulative distribution and with the auxiliary lf() wrapping function, respectively^13^.

[“Category-specific effects” refer to the unique relationships between the predictor variables and the ordinal variable at various levels of the response. This information is crucial for capturing potential subtleties in the relationship between dependent and independent variables. “Unequal variance models” were used to account for the distinct contributions of predictor variables in explaining the variance of the ordinal dependent variable^13^.]

As shown in [Table 3](#tbl-comparison_Exp1), the effects were consistent on a 7-point scale and the variances were similar between the two groups of Viewing Distance. Therefore, the model without category-specific effects and without unequal variance is favoured.

Table 3: Full model versus full models with category-specific effects or unequal variances.

| Model | LOO diff | st.error |
| --- | --- | --- |
| Full model | 0 | 0 |
| Full acat model | -2.0 | 1.7 |
| Full Uneq var model | -2.1 | 1.8 |

### Model evaluation

| 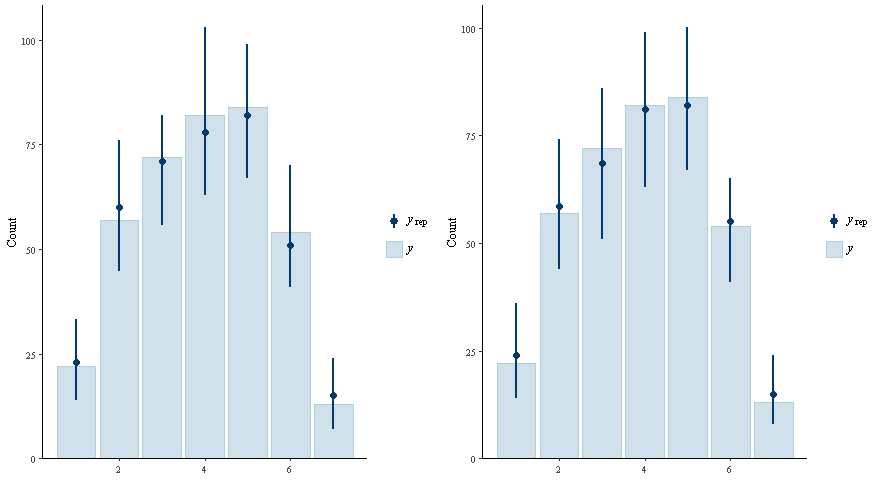  Figure 2: Posterior predictive check of the variables Line (left) and Distance (right) in Experiment 1. |
| --- |

The full model specifications were assessed using a posterior predictive check. This analysis compared the simulated data generated from the model with the actual data.

[Figure 2](#fig-ppcheckExp1) displays the 95% posterior credible intervals (indicated by blue lines) for each of the seven points of contentment for the variables Line (left) and Viewing Distance (right). This represents newly generated hypothetical data using posterior distribution parameters. The light bars represent the medians of the recorded ratings, whereas the actual predicted points are indicated by the dark blue dots. No major systematic discrepancies emerged in the data based on model predictions. This suggests that the model is an adequate fit for the data.

### Conditional effects

| 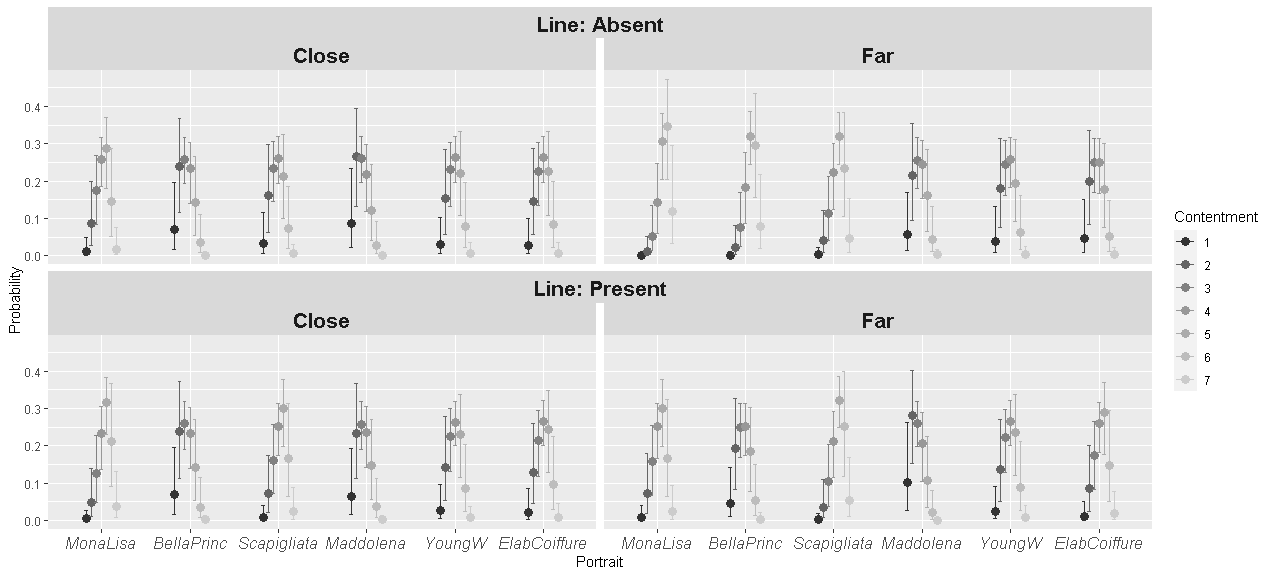  Figure 3: Conditional effects of each portrait in the two conditions of Viewing Distance (rows) and Line (columns) in Experiment 1. |
| --- |

[Figure 3](#fig-condeffectsExp1) illustrates the probability of expected ratings for each portrait, organised by Distance (columns) and Line (rows). As seen, lighter discs (indicating higher levels of contentment) are more probable at the Far viewing distance than at the Close viewing distance only for the three experimental portraits without the line. This difference does not appear in the other portraits, with lighter and darker discs equally spread between the two distances.

This suggests that the most probable ratings are higher from afar only in the unmodified portraits of Mona Lisa, Bella Principessa, and Scapigliata. In contrast, this difference is not observed when the same portrait had a line around the mouth. The control portrait ratings did not change with the viewing distance, regardless of the line.

### Results

**Effect of Viewing Distance.** On the latent opinion scale, participants rated portraits 0.91 SD more content from afar than from close [est. error = 0.44; l-95%CI = 0.06, u-95%CI = 1.77]. It can be concluded with a probability of at least 95% that the perceived contentment increased with distance. The following planned post hoc analysis assessed which portraits were affected by this manipulation.

**Effect of Line.** A line effect did not emerge. As will be seen in the planned post hoc analysis, this variable had a selective effect on portraits.

**Effect of Portrait.** There were few differences in the overall contentment of the portraits. The contrasts showed that on the latent opinion scale, participants rated Mona Lisa as having more contentment than La Bella Principessa [SD = 0.67; l-95% CI = 0.36 and u-95% CI = 0.94], Maddolena [SD = 1.13; est. error = 0.19; l-95%CI = 0.74, u-95%CI = 1.48], Young Woman [SD = 0.68; est. error = 0.18; l-95%CI = 0.33, u-95%CI = 1.05], and Elaborate Coiffure [SD = 0.60; est. error = 0.18; l-95%CI = 0.24, u-95%CI = 0.95]. Scapigliata was rated as more content than La Bella Principessa [SD = 0.49; l-95%CI = 0.79, u-95%CI = 0.20] and as less content than Maddolena [SD = -0.95; l-95%CI = -0.56, u-95%CI = -1.31], Young Woman [SD = -0.50; l-95%CI = -0.14, u-95%CI = -0.87] and Elaborate Coiffure [SD = -0.42; l-95%CI = -0.08, u-95%CI = -0.80]. Maddolena was also rated lower than Young Woman and Elaborate Coiffure [SD = -0.44; l-95%CI = -0.81, u-95%CI = -0.80 and SD = -0.52; l-95%CI = -0.88, u-95%CI = -0.15, respectively]. No other comparisons are noteworthy.

**Interaction between Viewing Distance and Line** The interaction effect between Viewing Distance and Line did not emerge. However, because predictors are conditional on the other factors in the model with which they interact, they do not provide comparisons that are interesting for this project, namely, the selective effect of Line and Viewing Distance in relation to each portrait. This comparison was evaluated in a subsequent post hoc analysis.

### Planned post hoc analysis

Table 4: Estimated difference in contentment between the Far and Close conditions of Viewing Distance in Experiment 1.

| **Distance** | **Portrait** | **Line** | **estimate** | **lower.HPD** | **upper.HPD** |
| --- | --- | --- | --- | --- | --- |
| Far - Close | Mona Lisa | Absent | 0.318 | 0.139 | 0.472 |
| Far - Close | Mona Lisa | Present | -0.074 | -0.333 | 0.173 |
| Far - Close | Bella Principessa | Absent | 0.514 | 0.304 | 0.718 |
| Far - Close | Bella Principessa | Present | 0.064 | -0.138 | 0.287 |
| Far - Close | Scapigliata | Absent | 0.307 | 0.069 | 0.544 |
| Far - Close | Scapigliata | Present | 0.134 | -0.101 | 0.380 |
| Far - Close | Maddolena | Absent | 0.060 | -0.127 | 0.279 |
| Far - Close | Maddolena | Present | -0.055 | -0.244 | 0.120 |
| Far - Close | Young Woman | Absent | -0.048 | -0.271 | 0.187 |
| Far - Close | Young Woman | Present | 0.007 | -0.235 | 0.252 |
| Far - Close | Elaborate Coiffure | Absent | -0.084 | -0.315 | 0.150 |
| Far - Close | Elaborate Coiffure | Present | 0.104 | -0.153 | 0.357 |

| 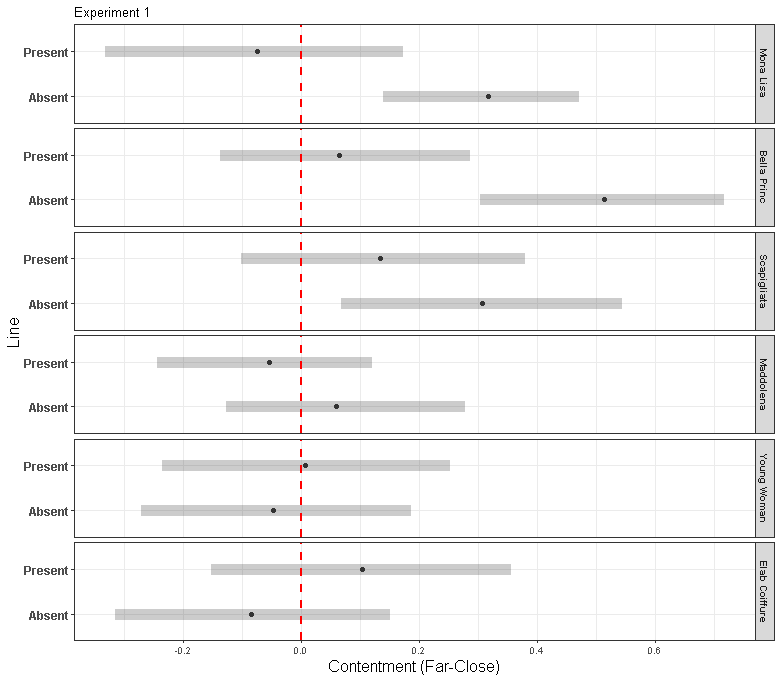  Figure 4: Medians and HDIs of the difference between the two viewing distances in Experiment 1. All HDI bars - but of Mona Lisa, Bella Principessa and Scapigliata without the line - overlap zero. |
| --- |

The estimated differences between the two distances for each portrait, as shown in [Table 4](#tbl-posthoctable_exp1), and the relative positions of the Highest Density Intervals (HDIs), as depicted in [Figure 4](#fig-posthocplot_exp1), indicate that while the perceived contentment in the three experimental portraits without the line increased with distance, it did not change in the conditions with the line.

The absence of any difference in viewing distance in the control portraits, regardless of the presence of the line, underscores the uniqueness of the effect. It also highlights how subtle elements in a portrait, such as the perceptual organisation of the Ambiguity Nuance, can significantly influence expression perception.

## **Experiment 2**

### Statistical Analysis

[Table 5](#tbl-descrexp2) shows the medians and median absolute deviations (MADs, in brackets) of the levels of contentment recorded in Experiment 2.

Table 5: Medians and MADs (in brackets) of the level of contentment recorded in Experiment 2.

| **Portrait** | **Ambiguity Smudge Pos** | **Close** | **Far** |
| --- | --- | --- | --- |
| Mona Lisa | Below | 4(1.48) | 3(0.74) |
| Mona Lisa | Above | 4(0.74) | 5(1.48) |
| Bella Principessa | Below | 4(1.48) | 2(0.00) |
| Bella Principessa | Above | 4(0.74) | 4.5(1.48) |
| Scapigliata | Below | 3.5(1.48) | 3(1.48) |
| Scapigliata | Above | 3(1.48) | 5(1.48) |

| 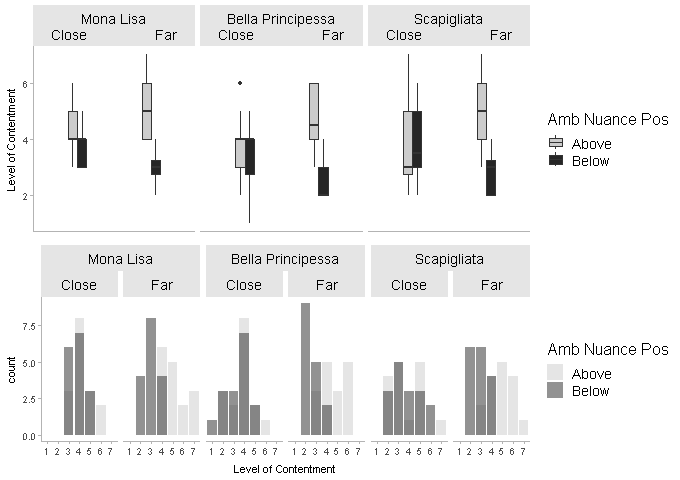  Figure 5: Top row: Box plot of contentment for the three portraits used in Experiment 2. Bottom row: Frequency of the seven ratings for the same portraits. |
| --- |

The first row of [Figure 5](#fig-resExp2revised) displays the box plot of the contentment level, and the second row shows the frequency of the seven ratings organised by Portrait and Viewing Distance of Experiment 2.

As seen in the first row of [Figure 5](#fig-resExp2revised), the light bars (indicating unmodified portraits) are higher in the Far condition than in the Close condition, whereas the dark bars (indicating portraits with the Ambiguity Nuance below the mouth) are lower in the Far condition than in the Close condition.

In the second row of [Figure 5](#fig-resExp2revised), the light bars (representing unmodified portraits) are more frequent in the lower range of contentment in the Close condition than in the Far condition. However, the opposite occurs for portraits with the Ambiguity Nuance below the mouth, as indicated by the dark bars.

This shows that while in the unmodified portraits, perceived contentment increased with distance, it decreased with distance when the Ambiguity Nuance was below the mouth.

### Modelling

A set of Bayesian linear mixed-effects models was used. [Table 6](#tbl-selection_Exp2) shows the results of the leave-one-out cross-validation method (LOO)^11^ used to select the model with the best predictive abilities.

Table 6: Model selection.

| **Model** | **LOO diff** | **st.error** |
| --- | --- | --- |
| Ambiguity Smudge Pos*Distance | 0.0 | 0.0 |
| Ambiguity Smudge Pos*Distance*Portrait | -3.4 | 3.8 |
| Ambiguity Smudge Pos*Portrait | -20.9 | 7.7 |
| Ambiguity Smudge Pos | -22.1 | 6.5 |
| Portrait | -47.1 | 8.9 |
| Empty model | -48.7 | 8.2 |
| Distance | -48.9 | 8.2 |
| Distance*Portrait | -50.4 | 8.9 |

As shown in [Table 6](#tbl-selection_Exp2), models that include Ambiguity Nuance Position and Viewing Distance variables have similar predictive abilities. This shows that the combined effects of Ambiguity Nuance Position and Distance were similar for the three portraits.

The leave-one-out (LOO) information criterion of the model that contains only the variables Ambiguity Nuance Position and Viewing Distance is 535.6 (standard error = 22.2). However, to examine the effects of Ambiguity Nuance Position and Viewing Distance on perceived contentment separately for each portrait, a full model that also includes Portrait must be used.

The LOO of the full model is 540.6 (standard error = 21.9), which is similar to the LOO of the model that contains Ambiguity Nuance Position and Viewing Distance only. The subsequent post hoc analysis is based on the full model.

As in Experiment 1, category-specific and unequal variance models were run using the acat() function instead of the cumulative distribution and with the auxiliary lf() wrapping function, respectively^13^. As shown in [Table 7](#tbl-comparison_Exp2), the effects were consistent on a 7-point scale and the variances were similar between the two groups of Viewing Distance. Therefore, the model without category-specific effects and without unequal variance is favoured.

Table 7: Full model versus full models with category-specific effects or unequal variances.

| **Model** | **LOO diff** | **st.error** |
| --- | --- | --- |
| Full Uneq var model | 0 | 0 |
| Full model | -0.6 | 1.6 |
| Full acat model | -1.4 | 1.6 |

### Model evaluation

| 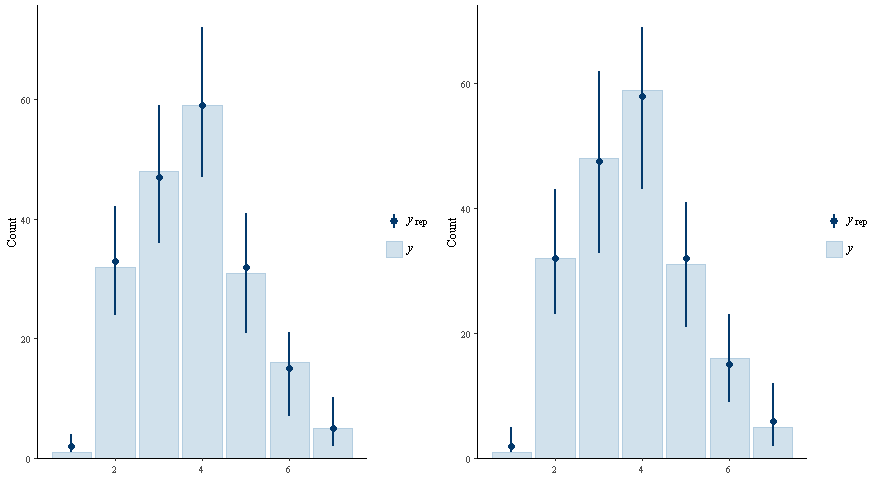  Figure 6: Posterior predictive check of the variables Ambiguity Nuance Position (left) and Distance (right) in Experiment 2. |
| --- |

The full model specifications were assessed using a posterior predictive check. [Figure 6](#fig-ppcheckExp2) displays the 95% posterior credible intervals (indicated by blue lines) for each of the seven points of contentment for the Ambiguity Nuance Position (left) and Viewing Distance (right). This represents newly generated hypothetical data using posterior distribution parameters. The light bars represent the medians of the recorded ratings, whereas the actual predicted points are indicated by the dark blue dots. No major systematic discrepancies emerged in the data based on model predictions. This suggests that the model is an adequate fit for the data.

### Conditional effects

| 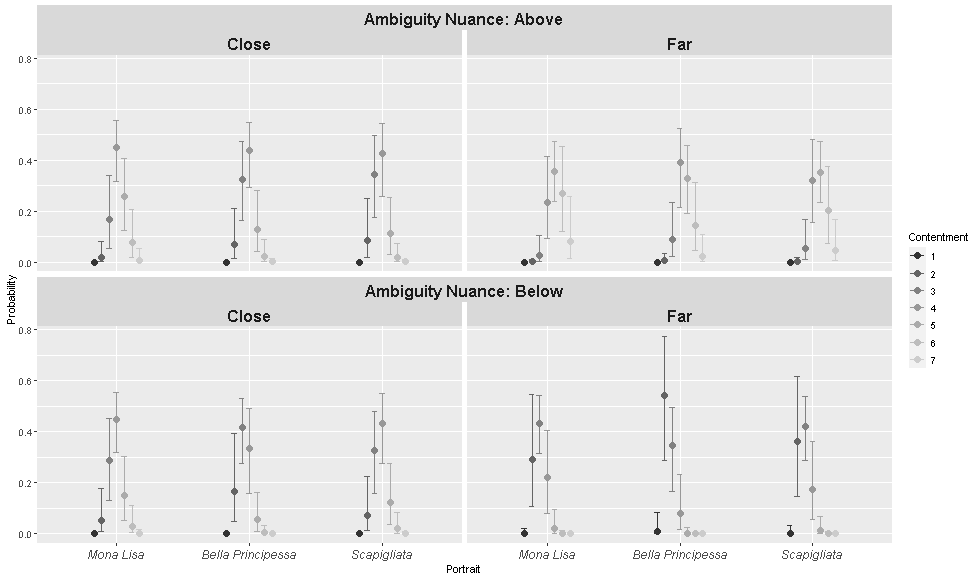  Figure 7: Conditional effects of each portrait in the two conditions of Viewing Distance (rows) and Ambiguity Nuance Position (columns) in Experiment 2. |
| --- |

[Figure 7](#fig-condeffectsExp2) illustrates the probability of expected ratings for each portrait, arranged by Distance (columns) and Ambiguity Nuance Position (rows). As seen, lighter discs (indicating higher levels of contentment) are more probable at the Far viewing distance than at the Close viewing distance only for the unmodified portraits. Conversely, darker discs (indicating lower levels of contentment) are more likely from afar in the portrait with the Ambiguity Nuance below the mouth.

This suggests that the most probable ratings are higher from afar only in the unmodified portraits. In contrast, they are lower from afar when the Ambiguity Nuance is below the mouth.

### Results

**Effect of Viewing Distance.** No effect of Viewing Distance emerged. The absence of a discernible effect of Viewing Distance on contentment is noteworthy, given the anticipated counteractive influences associated with different conditions of Ambiguity Nuance Position. The hypothesis posited that an increase in Viewing Distance would increase contentment when Ambiguity Nuance was above the mouth, but diminish contentment when the Ambiguity Nuance was below the mouth. The observed lack of a main effect of Viewing Distance implies a potential cancellation of these opposing influences. This cancellation is further supported by the significant interaction effect between Viewing Distance and Ambiguity Nuance (detailed in the subsequent interaction effect section).

**Effect of Ambiguity Nuance Position.** The point estimate of Ambiguity Nuance Position indicates that on the latent opinion scale, participants rated the portraits 1.18 SD [est. error = 0.17; l-95%CI = 0.85, u-95%CI = 1.51] more content under the condition Ambiguity Nuance Above.

**Effect of Portrait.** The point estimate of Portrait indicates that on the latent opinion scale, participants rated the Mona Lisa 0.46 SD [est. error = 0.19; l-95%CI = 0.11, u-95%CI = 0.83] more content than La Bella Principessa. Mona Lisa and Scapigliata were rated similarly in terms of contentment, as were Bella Principessa and Scapigliata.

**Interaction effect between Viewing Distance and Ambiguity Nuance Position.** On the latent opinion scale, the conditional effects on contentment of Ambiguity Nuance Position and Viewing Distance were different, with a 95% CI of this parameter not including zero [SD = 2.15, est. error = 0.33; l-95%CI = -1.5, u-95%CI = 2.80]. The selective effects of Ambiguity Nuance Position and Viewing Distance on each portrait were compared in the planned post hoc analysis.

### Planned post hoc analysis

Table 8: Estimated difference in contentment between the Far and Close conditions of Viewing Distance in Experiment 2.

| **Distance** | **Portrait** | **Ambiguity Nuance Pos** | **estimate** | **lower.HPD** | **upper.HPD** |
| --- | --- | --- | --- | --- | --- |
| Far - Close | Mona Lisa | Above | 0.348 | 0.158 | 0.484 |
| Far - Close | Mona Lisa | Below | -0.231 | -0.432 | -0.057 |
| Far - Close | Bella Principessa | Above | 0.373 | 0.116 | 0.617 |
| Far - Close | Bella Principessa | Below | -0.105 | -0.232 | -0.009 |
| Far - Close | Scapigliata | Above | 0.498 | 0.264 | 0.718 |
| Far - Close | Scapigliata | Below | -0.203 | -0.385 | -0.042 |

| 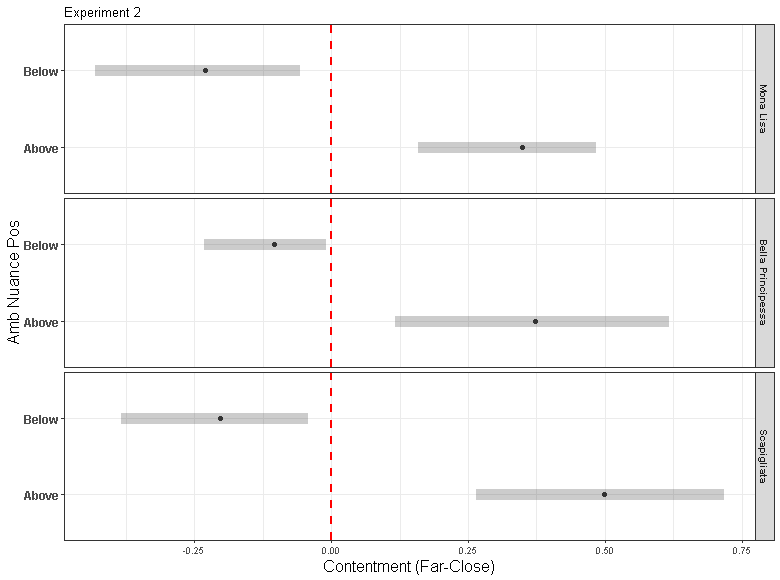  Figure 8: Medians and HDIs of the difference between the two viewing distances in Experiment 2. None of the HDI bars overlap zero. |
| --- |

The estimated differences between the two distances for each portrait ([Table 8](#tbl-posthoctable_exp2)) and the relative positions of the HDI ([Figure 8](#fig-posthocplot_exp2)) indicate that the contentment was higher in the unmodified portraits when viewed from afar than when viewed from up close. However, it was lower from afar than from close when the Ambiguity Nuance was below the mouth.

### Ambiguity Nuance viewed from close

This analysis focused on the effects of the Ambiguity Nuance Position under the Close condition of the Viewing Distance. The point estimate revealed that on the latent opinion scale, there was no significant difference in the conditional effect, as evidenced by a 95% CI for the estimated parameter that included zero (SD = 0.47 [est. error = 0.37); l-95%CI = -1.19, u-95%CI = 0.26]. As in the previous analysis, a post hoc comparison was extracted using the emmeans package^14^.

| 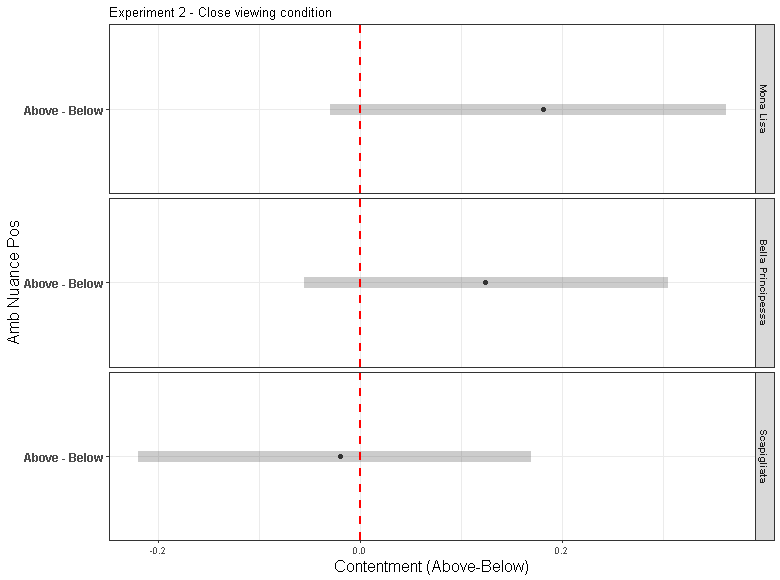  Figure 9: Medians and HDIs of the difference in contentment between the Above and Below Ambiguity Nuance conditions from close. All HDI bars overlap zero. |
| --- |

Table 9: Estimated difference in contentment between the Above and Below conditions of Ambiguity Nuance Position from Close.

| **Ambiguity Nuance Pos** | **Portrait** | **estimate** | **lower.HPD** | **upper.HPD** |
| --- | --- | --- | --- | --- |
| Above - Below | Mona Lisa | 0.181 | -0.030 | 0.362 |
| Above - Below | Bella Principessa | 0.124 | -0.055 | 0.305 |
| Above - Below | Scapigliata | -0.020 | -0.220 | 0.169 |
|  |  |  |  |  |

As shown in [Table 9](#tbl-posthoctable_exp2b) and [Figure 9](#fig-posthocplot_exp2b), the HDI estimate of the post hoc analysis for all portraits includes zero. This indicates that in the Close condition, both modified and unmodified portraits elicited similar levels of contentment. These findings suggest that when the boundary between the mouth and Ambiguity-Nuance is distinguishable, it does not affect contentment. Therefore, it can be concluded that the change in expression is not solely due to the presence of the Ambiguity Nuance. Rather, it is the perceptual organisation applied to the Ambiguity Nuance, which plays a significant role. This finding emphasizes the influential role of perceptual organisation in the perception of expression in these portraits.

# References:

1. Carpenter, B. *et al.* [Stan: A probabilistic programming language](https://doi.org/10.18637/jss.v076.i01). *Journal of statistical software* **76**, 1–32 (2017).

2. Bürkner, P.-C. [Brms: An r package for Bayesian multilevel models using stan](https://doi.org/%2010.18637/jss.v080.i01). *Journal of statistical software* **80**, 1–28 (2017).

3. Bürkner, P.-C. Advanced Bayesian multilevel modeling with the r package brms. *arXiv preprint arXiv:1705.11123* (2017) doi:[10.32614/rj-2018-017](https://doi.org/10.32614/rj-2018-017).

4. R Core Team. [*R: A language and environment for statistical computing*](https://www.R-project.org/). (R Foundation for Statistical Computing, 2023).

5. Liddell, T. M. & Kruschke, J. K. [Analyzing ordinal data with metric models: What could possibly go wrong?](https://doi.org/10.1016/j.jesp.2018.08.009%20) *Journal of Experimental Social Psychology* **79**, 328–348 (2018).

6. Gelman, A. & Rubin, D. B. [Inference from iterative simulation using multiple sequences](https://doi.org/10.1214/ss/1177011136). *Statistical science* **7**, 457–472 (1992).

7. Box, G. E. P. & Tiao, G. C. *Bayesian inference in statistical analysis*. (Wiley, 1992).

8. Chen, M.-H., Shao, Q.-M. & Ibrahim, J. G. Computing Bayesian credible and HPD intervals. in *Monte Carlo Methods in Bayesian Computation* (eds. Chen, M.-H., Shao, Q.-M. & Ibrahim, J. G.) 213–235 (Springer, 2000). doi:[10.1007/978-1-4612-1276-8_7](https://doi.org/10.1007/978-1-4612-1276-8_7).

9. Hespanhol, L., Vallio, C. S., Costa, L. M. & Saragiotto, B. T. [Understanding and interpreting confidence and credible intervals around effect estimates](https://doi.org/10.1016/j.bjpt.2018.12.006). *Brazilian Journal of Physical Therapy* **23**, 290–301 (2019).

10. Kruschke, J. K. & Liddell, T. M. [Bayesian data analysis for newcomers](https://doi.org/10.3758/s13423-017-1272-1). *Psychonomic bulletin & review* **25**, 155–177 (2018).

11. Vehtari, A., Gelman, A. & Gabry, J. [Practical Bayesian model evaluation using leave-one-out cross-validation and WAIC](https://doi.org/10.1007/s11222-016-9696-4). *Statistics and computing* **27**, 1413–1432 (2017).

12. Akaike, H. Information theory and an extension of the maximum likelihood principle. in *Selected papers of Hirotugu Akaike* 199–213 (Springer, 1998). doi:[10.1007/978-1-4612-0919-5_38](https://doi.org/10.1007/978-1-4612-0919-5_38).

13. Bürkner, P.-C. & Vuorre, M. [Ordinal regression models in psychology: A tutorial](https://doi.org/10.1177/2515245918823199). *Advances in Methods and Practices in Psychological Science* **2**, 77–101 (2019).

14. Lenth, R. V. [*Emmeans: Estimated marginal means, aka least-squares means*](https://CRAN.R-project.org/package=emmeans). (2022).

15. Kruschke, J. K. [Bayesian analysis reporting guidelines](https://doi.org/10.1038/s41562-021-01177-7%20). *Nature Human Behaviour* **5**, 1282–1291 (2021).
